# Ground state and stability of the fractional plateau phase in metallic Shastry–Sutherland system TmB_4_

**DOI:** 10.1038/s41598-021-86353-5

**Published:** 2021-03-25

**Authors:** Matúš Orendáč, Slavomír Gabáni, Pavol Farkašovský, Emil Gažo, Jozef Kačmarčík, Miroslav Marcin, Gabriel Pristáš, Konrad Siemensmeyer, Natalya Shitsevalova, Karol Flachbart

**Affiliations:** 1 Institute of Experimental Physics, SAS, Watsonova Str. 47, 04001 Košice, Slovakia; 2 Helmholtz-Zentrum Berlin, Hahn-Meitner Platz 1, 14109 Berlin, Germany; 3 Institute for Problems of Materials Science, NASU, Krzhyzhanovsky Str. 3, Kyiv, 03142 Ukraine

**Keywords:** Materials science, Physics

## Abstract

We present a study of the ground state and stability of the fractional plateau phase (FPP) with M/M_sat_ = 1/8 in the metallic Shastry–Sutherland system TmB_4_. Magnetization (M) measurements show that the FPP states are thermodynamically stable when the sample is cooled in constant magnetic field from the paramagnetic phase to the ordered one at 2 K. On the other hand, after zero-field cooling and subsequent magnetization these states appear to be of dynamic origin. In this case the FPP states are closely associated with the half plateau phase (HPP, M/M_sat_ = ½), mediate the HPP to the low-field antiferromagnetic (AF) phase and depend on the thermodynamic history. Thus, in the same place of the phase diagram both, the stable and the metastable (dynamic) fractional plateau (FP) states, can be observed, depending on the way they are reached. In case of metastable FP states thermodynamic paths are identified that lead to very flat fractional plateaus in the FPP. Moreover, with a further decrease of magnetic field also the low-field AF phase becomes influenced and exhibits a plateau of the order of 1/1000 M_sat_.

## Introduction

Quantum spins with AF coupling on frustrating lattices have attracted widespread interest due to the discovery of new types of complex quantum ground states as e.g. spin ice^1^, quantum spin liquid-like states^2^, and fractional magnetization plateaus on the Shastry–Sutherland lattice^3^, among them on SrCu_2_(BO_3_)_2_^4–9^ as well as on the family of rare earth tetraborides, REB_4_ (RE = Tm, Er, Ho, Dy, Tb, Gd, Sm, Nd)^10–25^. Even if they share the same frustrated lattice, the phase diagrams of the magnetic REB_4_ compounds differ from SrCu_2_(BO_3_)_2_. In the insulator SrCu_2_(BO_3_)_2_ the exchange interaction is of the Heisenberg type while the REB_4_ magnets are metals and it is generally supposed that the AF exchange interaction between the magnetic moments is caused by the long range Ruderman–Kittel–Kasuya–Yosida (RKKY) type mediated by conduction electrons. Probably the most investigated among REB_4_ compounds is TmB_4_ which orders antiferromagnetically at *T*_*N1*_ = 11.7 K. It has attracted attention for its rich magnetic phase diagram which is strongly biased by crystal field effects at the Tm^3+^ ion sites that lift the degeneracy of the *J* = 6 multiplet and lead to a *M*_*J*_ =  ± 6 ground state doublet^15,26–30^. TmB_4_ thus exhibits strong Ising anisotropy where the saturation field along the easy *c* axis is at least 10 times smaller than in the perpendicular *a-b* plane. In the ordered phase so far three different phases are well established: The low field AF phase consists of an AF arrangement of dimers, where the dimer spins are ferromagnetic. The HPP with *M/M*_*sat*_ = ½ in fields above about 17.5 kOe consists of ferromagnetic stripes of 4 lattice units size embedded in a sea of antiferromagnetic dimers. These phases are separated by the so called FPP, where magnetization plateaus with magnetization values of 1/7, 1/8 1/9 *M*_*sat*_ and other fractions are observed. The structure of this phase resembles ferromagnetic domain walls between antiferromagnetic dimers, but with a strictly regular arrangement over 7, 8 or 9 unit cells^15,28^.

The microscopic reason for this complex long ranged ordering scenario is still under debate, and one may mention higher order interactions, Chern–Simons fields or other topological reasons that can occur in this Archimedean lattice^31–33^.

At the core of the discussion is the question whether the FPP states are thermodynamic stable or of a dynamic origin, which lie in the transition from the AF to HP phase. The idea behind that is that the phases are structurally very different and a huge amount of spin flips is required for the phase transition. At low temperature, the strong Ising nature of TmB_4_ may well freeze the transformation dynamics between phases, leading to unconventional dynamics as also has been discussed for SrCu_2_(BO_3_)_2_^32,34–36^. Very recently in Ref.^37^ the 1/8 fractional plateau, which appears in the FPP between 14 and 17.5 kOe, has been proposed to be of dynamical origin. Their observations lead the authors to conclude that the 1/8 FP is not a thermodynamically stable state, but rather a metastable variant of the AF state created on approaching the phase boundary to the HPP. Their result suggested that the observed fractional magnetization should not be considered as a stable state of the system and that the plateau phases need to be reconciled with the complete phase diagram. Similarly, by means of neutron- and X-ray-scattering experiments and magnetization measurements, it was shown in Ref.^25^ that the 1*/*8 FP is metastable, arising because the spin dynamics is frozen below *T* ≈ 4*.*5 K.

Here, we present a study of stability of the FPP states as a function of time, magnetic field and temperature change based on magnetization and ac-calorimetric measurements. It turns out that in contrast to earlier measurements FP states can well be observed after cooling in constant field, therefore they must be considered as thermodynamic stable. Remarkably, the hysteresis observed in reaching FP states after zero field cooling that led to the conjecture of dynamic states is always present. The magnetization and specific heat data here suggest an apparent connection between the FPP and HPP, which is related to the sample history.

It is observed that after reaching the low-field AF phase by decreasing *H* from the HPP (across the FPP), also the AF phase itself becomes influenced and does not exhibit the same properties as in its pristine state obtained after zero-field cooling. These modifications appear below 4 K in fields between 6 and 14 kOe, and exhibit FPs which are about two orders of magnitude smaller than those in the FPP. Moreover, it appears that the FPP depends strongly on the thermodynamic history which includes changes of magnetic field as well as temperature. Here we identify thermodynamic paths which lead to very flat fractional plateaus with a wide range of magnetization values between 1/8 *M*_*sat*_ to 1/80 *M*_*sat*_ at 2 K and 16 kOe. The results bear important impact on future experimental investigations of this frustrated system as well as on the theoretical modeling of the magnetization processes in systems with Shastry–Sutherland lattice.

## Experimental

Magnetization measurements were performed in parallel at Košice and in Berlin on two samples with different size and demagnetization factor: A flat one (1 × 1 × 0.25 mm^3^) was used for heat capacity and magnetization measurements. Magnetization experiments were repeated using a long sample (1 × 1 × 4 mm^3^). Both samples were cut from the same single crystalline rod. Magnetization data were measured using commercial DC and VSM SQUIDs. These parallel investigations led to identical results. The heat capacity was measured with a high precision ac-calorimetry method, using a light emitting diode as heat source. This very fast method provides accurate relative values of the specific heat (details are in Ref.^38^). The original TmB_4_ rod was grown by an inductive, crucible-free zone melting method, its residual resistivity ratio was larger than 20, documenting its high quality.

## Results and discussion

**Figure 1:**
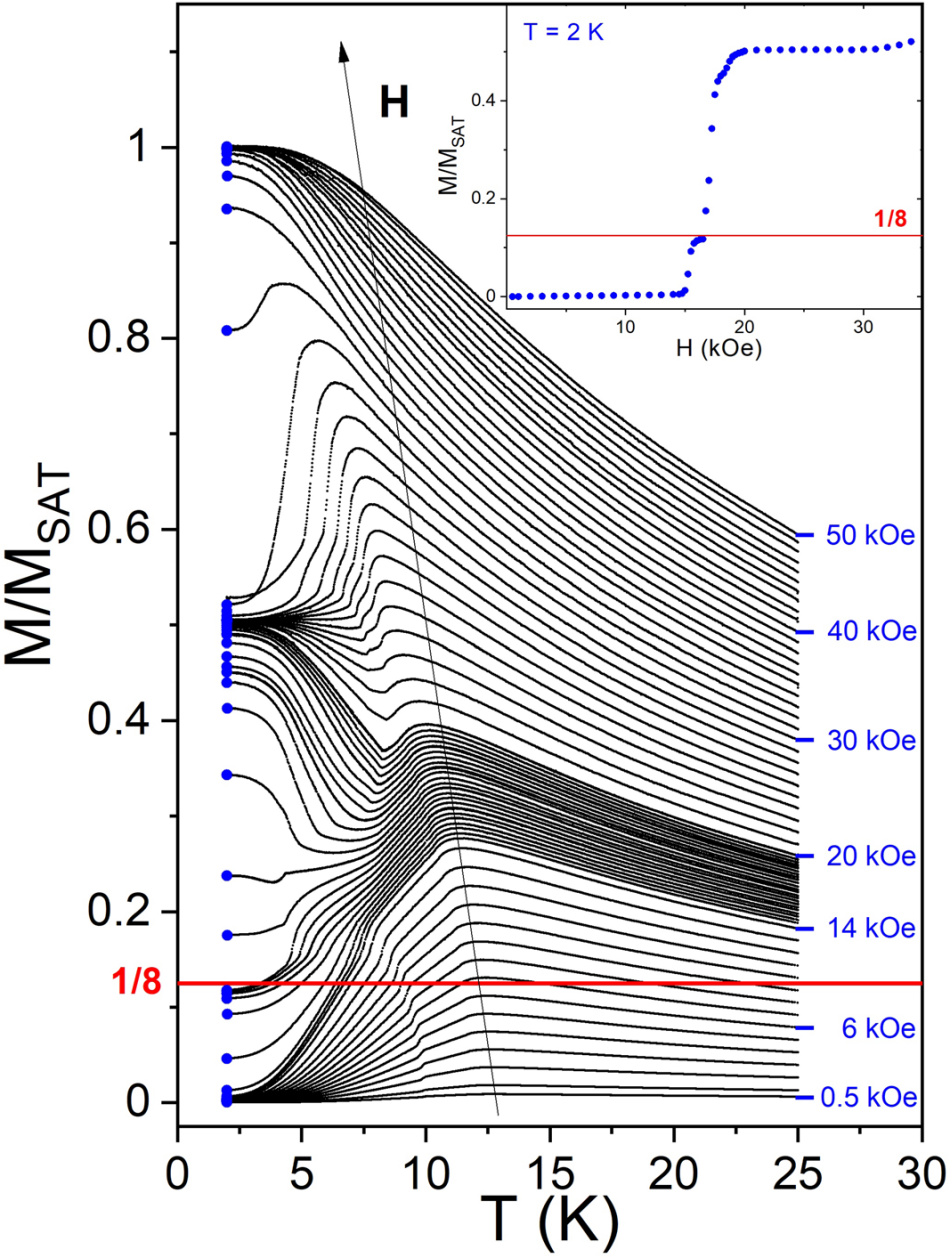
Temperature dependence of magnetization *M* on cooling from 25 K (paramagnetic state) to 2 K (ordered state) in various constant magnetic fields. The inset shows the derived field dependence of *M* at 2 K which exhibits a distinct thermodynamically stable 1/8 fractional plateau (sFP).

First, magnetization (*M*) measurements were performed when the sample was in a constant magnetic field cooled slowly from the paramagnetic state at 25 K to the ordered state at 2 K (see Fig. 1). In this procedure the system finds at given field its thermodynamically most favorable (stable) low-temperature ground state. From these data the field dependence of magnetization at 2 K is obtained. It is displayed in the inset of Fig. 1 and exhibits a clear FP with *M/M*_*sat*_ ≈ 1/8. This, in contrast to earlier investigations, shows that these FPP states must be considered as thermodynamically stable (here denoted as stable fractional plateaus, sFP).

**Figure 2:**
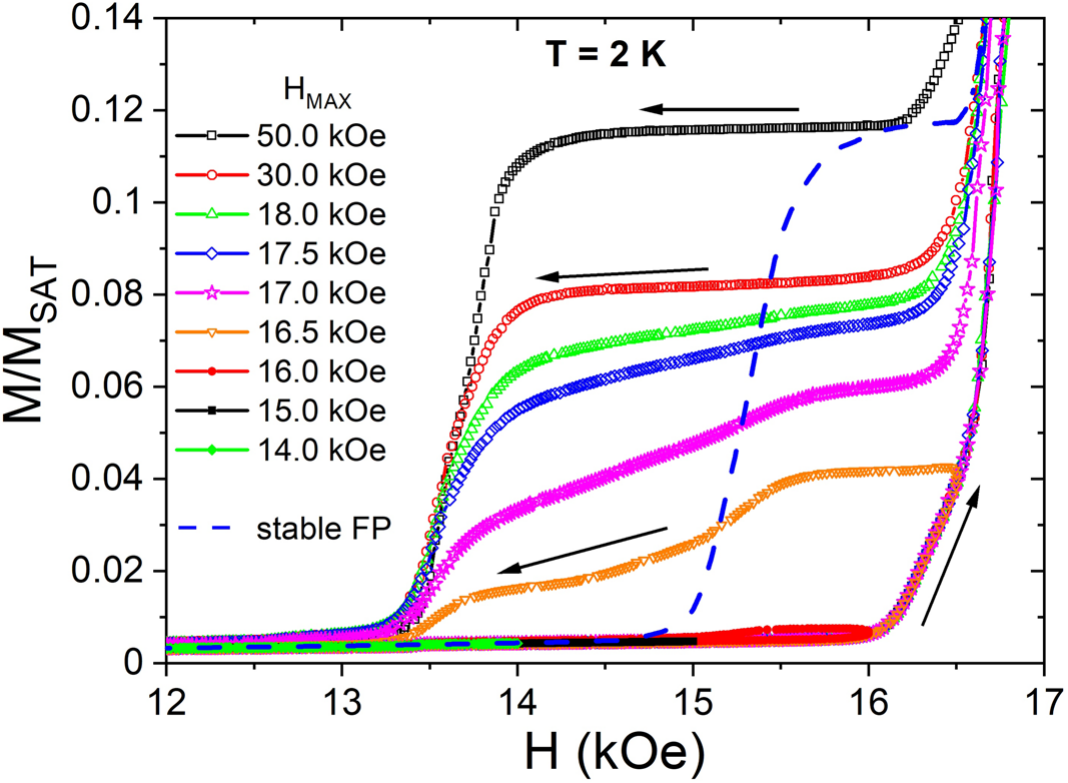
Field dependence of the magnetization *M* at 2 K after zero-field cooling. With gradually increasing field hysteresis loops can be observed. Note that distinct fractional plateaus develop only after *H* first reaches values close to the half plateau phase (for fields of about 16.5 kOe and above). The dashed line shows the stable fractional plateau (sFP) obtained after cooling in constant field (see Fig. 1).

The hysteresis observed in magnetization processes starting from low temperature appears contradictory to the presence of an equilibrium state. To clarify this the field dependence of the magnetization *M*(*H*) was measured after zero field cooling (Fig. 2), i.e. when starting from the pristine low-field AF phase. One can see that in this case a fractional plateau with *M/M*_*sat*_ = 1/8 develops on demagnetization only after *H* first reaches values close to the HPP (for fields of about 16.5 kOe and above). For *H* < 16.5 kOe the *M*(*H*) dependence shows only hysteresis loops which possibly are related to the time dependence of the magnetization (see Supplementary Fig. S1). A comparison between the 1/8 fractional plateau obtained in this way (we can denote it as metastable fractional plateau, mFP) and the one obtained after cooling in constant field (sFP) is shown in Fig. 2. It turns out that the mFP towards low fields is much wider than the sFP. In both cases, however, they lie immediately below the HPP.

The HPP consists of ferromagnetic (FM) stripes of 4 lattice units widths^15^ and it appears that with decreasing field this structure survives in a modified form with narrower FM stripes in the FPP^15,28^. That raises the conjecture that the FPP mediates the transition from the low-field AF phase to the HPP. The structure of the AF and HP phase indeed cannot be easily transformed into each other because a huge amount of spin flips is required. TmB_4_ is a strong Ising system with a spin gap of ~ 10 meV with suppressed dynamics at low temperature. It is, however, conceivable that at low temperature complex dynamic processes occur and manifest themselves in the FPP and corresponding structures, forming a thermodynamically stable ground state on slow cooling in a constant magnetic field. However, after zero-field cooling to low temperature the spin system is unable to form the FPP from the AF phase with increasing field as the dissimilarity between magnetic structures of the pristine low-field AF phase and the ordered FPP is big and the spin dynamics is frozen. In this case the FPP develops only after first reaching the HPP. Both phases share the stripe-like magnetic structure and the size of the magnetic unit cell, i.e. magnetic structure transformations between them could be easier comparing to the transition to the AF phase. It is remarkable that the hysteresis between the AF and FP phase is seen only in the first magnetization process, it is absent in all following magnetizations (e.g. Ref.^15^). This suggests that remnants of the field structure survive in the AF phase.

**Figure 3:**
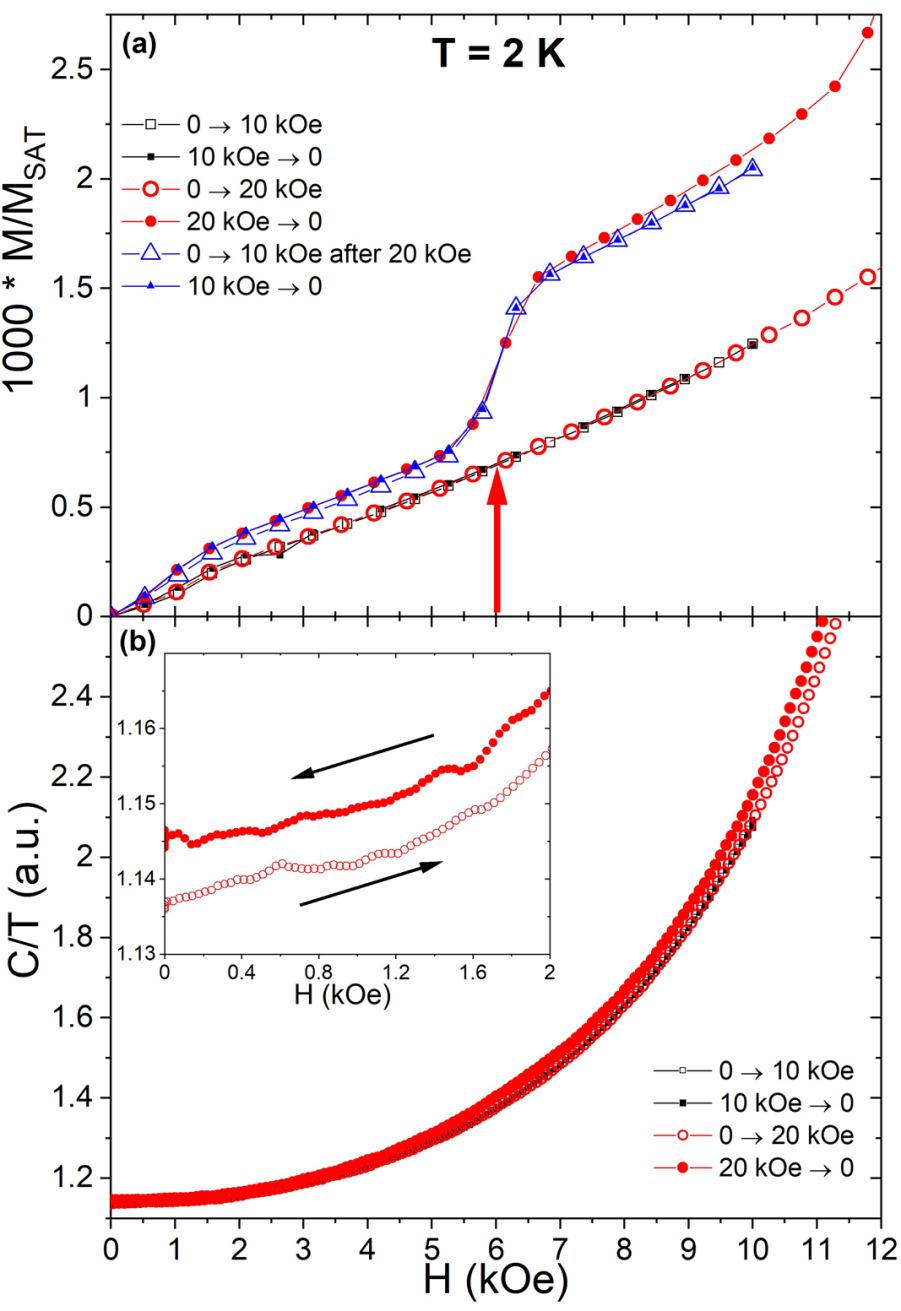
History dependence of the pristine low-field AF Néel phase after magnetizing the system up (and down) to 10 kOe and to 20 kOe in *M*(*H*) (**a**) and *C/T* (**b**). The observed change (jump) of magnetization corresponds to ~ 1/1000 *M*_*sat*_.

Some “remnants” of the FPP states also seem to survive if *H* is lowered below 14 kOe into the low-field AF Néel phase. To show this the following experimental protocol was used (same for *M* and *C/T*): the system was first cooled in zero field from 30 to 2 K, then the field was swept in following sequence: 0 kOe → 10 kOe → 0 kOe, then 0 kOe → 20 kOe → 0 kOe (Fig. 3). One can see that in magnetization measurements (Fig. 3a) a new very small jump emerges after the system was magnetized to 20 kOe (reaching the HPP). The value of this jump is about 1/1000 *M*_*sat*_ and it appears in fields higher than ~ 6 kOe. An additional 0 kOe → 10 kOe → 0 kOe sequence was performed to confirm the reproducibility of the new magnetization jump in the Néel phase after magnetizing into the HPP. As for the mFP, the hysteresis is absent after the first magnetization into the HPP.

Heat capacity measurements have proven the Néel phase modification, the *C/T* values and the corresponding entropy increases after the system was magnetized to 20 kOe (Fig. 3b). These results point to a change of the Néel phase texture after the HPP is reached. Nevertheless, in the heat capacity there is no anomaly at 6 kOe while in magnetization at this field a new jump emerges. This might relate to the fact that this jump has a very small magnetization value (1/1000 *M*_*sat*_), which means that for every 500 spins pointing down there are 501 spins pointing up for *H* > 6 kOe compared to 500:500 ratio for *H* < 6 kOe. The effect is therefore very subtle, well seen in sensitive magnetization measurements using a SQUID magnetometer. Such small changes in the spin arrangement may be below the resolution limit of the ac-calorimetry so that only a shift to higher *C*/*T* values remains observable in the AF phase.

**Figure 4:**
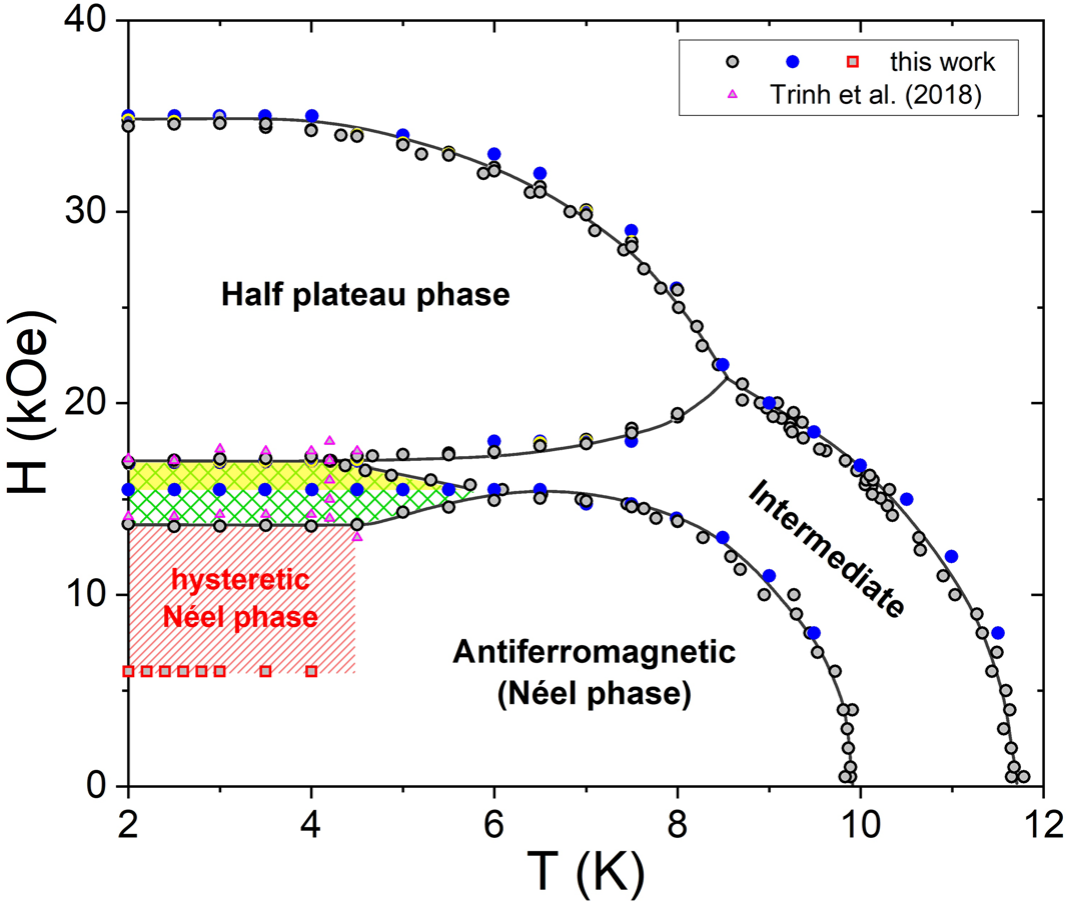
Phase diagram of TmB_4_ showing the part with stable fractional plateaus (sFP), yellow area above the blue points, and the part with metastable fractional plateaus (mFP), whole hatched green area, of the FPP and the ”hysteretic” part of the AF Néel phase with ~ 1/1000 *M*_*sat*_ magnetization jumps (red hatched area above the red points). Note that the sFP area can be reached by cooling in constant field directly from the intermediate phase. Black, blue and red points were determined from various magnetization measurements, magenta points (triangles) were taken from Ref.^37^.

To see where the new small magnetization jumps stabilize in the *H*–*T* phase diagram, the previous experimental procedure (plotted in Fig. 3a) was performed also at temperatures up to 5 K (see Supplementary Fig. S2). Figure 4 shows the resulting *H*–*T* phase diagram of TmB_4_, which includes the part of sFP (yellow area) and the part of mFP (full area hatched in green) of the FPP as well as the “hysteretic” part of the AF Néel phase, which contains the very small ~ 1/1000 *M*_*sat*_ magnetization jumps. Blue points of phase diagram were obtained from field sweeps reconstructed from detailed temperature dependences (see inset of Fig. 1).

**Figure 5:**
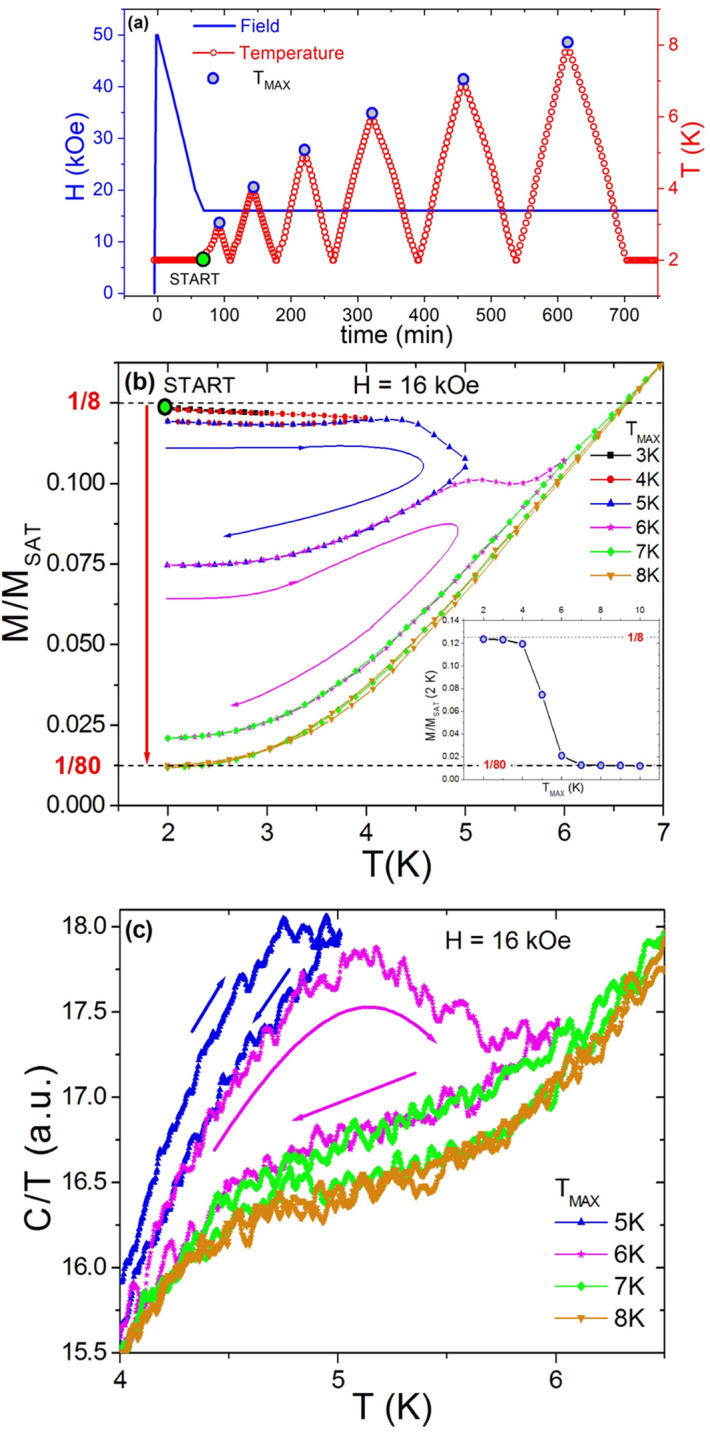
Field and temperature protocol (**a**) of annealing cycles, and the resulting magnetization (**b**) and heat capacity (**c**) changes. The big green point in (**a**) is the starting point in the FPP obtained by demagnetization from 50 to 16 kOe at 2 K, the annealing cycles are marked by various colors. The final *M/M*_*sat*_ values at 2 K and 16 kOe after annealing are shown in the inset of (**b**), *T*_*max*_ represents the maximum annealing temperature.

To investigate the stability of the FPP states further, temperature hysteresis experiments were performed. Here, first a magnetization curve and the corresponding heat capacity was measured as a reference (see Supplementary Fig. S3). The obtained *C/T* vs. *H* and *M* vs. *H* dependence in the vicinity of FPP region are in good agreement with those obtained in Ref.^37^. The temperature hysteresis experiments started at low temperature in the saturated state (at 2 K in field of 50 kOe), then *H* was decreased isothermally to 16 kOe. At this point (2 K, 16 kOe) the system is in the FPP with a *M/M*_*sat*_ = 1/8 metastable fractional plateau (mFP). Subsequently at 16 kOe a few thermal cycles (the protocol of these *T*-cycles is shown in Fig. 5a) with increasing maximum temperature *T*_*max*_ of the cycles were performed. This process corresponds to annealing and the resulting dependence of *M/M*_*sat*_ and *C/T* is plotted in Fig. 5b,c. As can be seen in Fig. 5b, with each annealing *T*-cycle a decrease of *M/M*_*sat*_ at 2 K is observed. Finally, after annealing to *T*_*max*_ above 6 K, *M/M*_*sat*_ values smaller than 1/80 can be observed. Corresponding *C/T* dependencies are in Fig. 5c, they exhibit a hysteretic behavior between 5 and 7 K, and point to a decrease of *C*/*T* with increasing *T*_*max*_. The final *M/M*_*sat*_ values of thermal cycles are plotted in the inset of Fig. 5b. These results appear to be consistent with the observations in Ref.^25^, where in this part of the phase diagram many long-ranged ordered phases with different propagation vectors are observed, in particular when the applied field drives the system from one plateau phase to another. One should also mention that interesting field and temperature dependent effects are observed in other REB_4_ compounds, e.g. in NdB_4_^23^ the temperature dependent susceptibility on cooling down to 0.5 K shows an unexpected transition, the system returns to the zero field antiferromagnetic state from a higher-temperature ferrimagnetic state.

The *M/M*_*sat*_ drop between 4 and 6 K resembles a phase transition, a point of disintegration of the FPs. At this drop at increasing temperature the fractional plateau states disintegrate, therefore on the way back (at lowering temperature) can the new fractional plateaus have already a much smaller *M/M*_*sat*_ value. This behavior is in agreement with^37^ where sharp ordering/disordering features in the *C/T* vs. *T* dependence at 4.2 K were observed in the field region around the 1/8 FPP. Thus, the magnetization within the mFP seems to be very sensitive to annealing, especially when the annealing temperature exceeds ~ 4 K. It turns out that the hysteresis of FPs which in previous work (see e.g. Refs.^15,30^) was located between about 14 and 17.5 kOe and between 0 and about 8 K, is limited only to the temperature range up to ~ 4 K as is shown also in Ref.^37^. At higher temperature, in the isothermal *M*(*H*) dependence, no fractional plateaus, but only hysteresis features can be observed (see e.g. Ref.^27^). However, it should be noted that in case of sFP under the annealing process no change of *M/M*_*sat*_ is observed, which underlines the thermodynamic stability of these plateaus. In this case namely the sFP is heated up into the intermediate phase and on the way back it goes through the same path as during the original cooling in constant field.

## Conclusions

Magnetization measurements on cooling in constant magnetic field show that the FPP states obtained in this way should be considered as a stable ground state of TmB_4_. On the other hand, these states after zero-field cooling and a subsequent increase of magnetic field appear in part as metastable states because then the fractional plateaus are observed only after the magnetization first reaches values close to the HPP.

Thus, it appears that in the same area of the phase diagram both of these states, the stable (sFP) and the metastable (mFP) states can be observed, depending on the way how they were reached. As these states exhibit different properties, the observation of such contradictory phenomena in a frustrated system is quite unique. The difference between the stable and metastable FPP states must relate to their different microscopic magnetic structure. The nature of such differences at present is speculative, e.g. one could think of special domain distributions that become favored by reaching the HPP.

The strong Ising anisotropy and large structural differences between the AF Néel phase suppress the relaxation from one phase to another. The FPP appears to mediate the transition between the AF and the HP phase as its magnetic structure exhibits features of both phases in form of FM stripes and AF dimers. It is interesting to note the HPP and FPP share the same lattice unit of 8 chemical cells, together with the intermediate phase^15^ at higher temperature (see Fig. 4). This large magnetic unit cell seems to play an important role for the formation of FP. We have shown here that sFP form on cooling in constant field, but only from the intermediate phase. Even more subtle changes of magnetization appear below 4 K in a field stripe between 6 and 14 kOe, and manifest themselves as ~ 1/1000 *M*_*sat*_ magnetization jumps. Their relation to structural changes needs further investigation.

Combining the structures of various magnetic phases obtained in Refs.^15,28^ with the results presented here leads to following hypothesis for the mechanism of hysteresis: In the HPP large AF and FM areas are present, characterized by wave vector *q* = 1/8, which is also characteristic for the FPP. It is therefore not far-fetched to assume that some of boundaries between FM and AF areas in the HPP persist in the FPP, simply because the necessary number of spin flips becomes reduced. One can consider that such footprints of the HPP persist also in the low-field AF phase, although the microscopic mechanism of such watermarks is not clear at present. And one can speculate that it is the absence of such watermarks in the virgin AF state which prevents the FPP formation in the first magnetization process, which also directly relates to very large time constants in the FPP formation. Thus, the hysteresis of FPP states strongly depends on the thermodynamic history. In this regard thermodynamic paths have been found which enable to obtain very flat fractional plateaus.

All these findings can have an important input for further theoretical modeling of this metallic frustrated system based on the Shastry–Sutherland lattice.

## Supplementary Information

Supplementary Information.
