# Supplementary material for: Ground state and stability of the fractional plateau phase in metallic Shastry–Sutherland system TmB4
Source: Sci Rep. 2021 Mar 25;11:6835. doi: 10.1038/s41598-021-86353-5 (PMC7994547; doi:10.1038/s41598-021-86353-5)
Supplement: Supplementary file 1 — Supplementary Information. [file 41598_2021_86353_MOESM1_ESM.docx]

**Supplemental Material**

**Ground state and stability of the fractional plateau phase**

**in metallic Shastry-Sutherland system TmB_4_**

Matúš Orendáč, Slavomír Gabáni, Pavol Farkašovský, Emil Gažo,

Jozef Kačmarčík, Miroslav Marcin, Gabriel Pristáš, Konrad Siemensmeyer,

Natalya Shitsevalova, Karol Flachbart

**Results and discussion**

We have started with investigation of the relationship between the pristine low-field AF phase and the fractional plateau phase (FPP). In this case was the sample first heated up to 30 K and subsequently zero-field cooled to 2 K. Then the field *H* was with a sweep rate of 200 Oe/s increased to various fields between 15 and 17.25 kOe (to reach FPP states), and the time dependence of magnetization (*M*) measured (Figure S1). The observed dependencies show a marked increase of *M* with time only for *H* > 15 kOe. But also for *H* = 16.5 kOe, close to the half plateau phase (HPP) field, the time dependence of *M/M_sat_* does not reach the expected FP value of ~1/8, not even in a few hours.


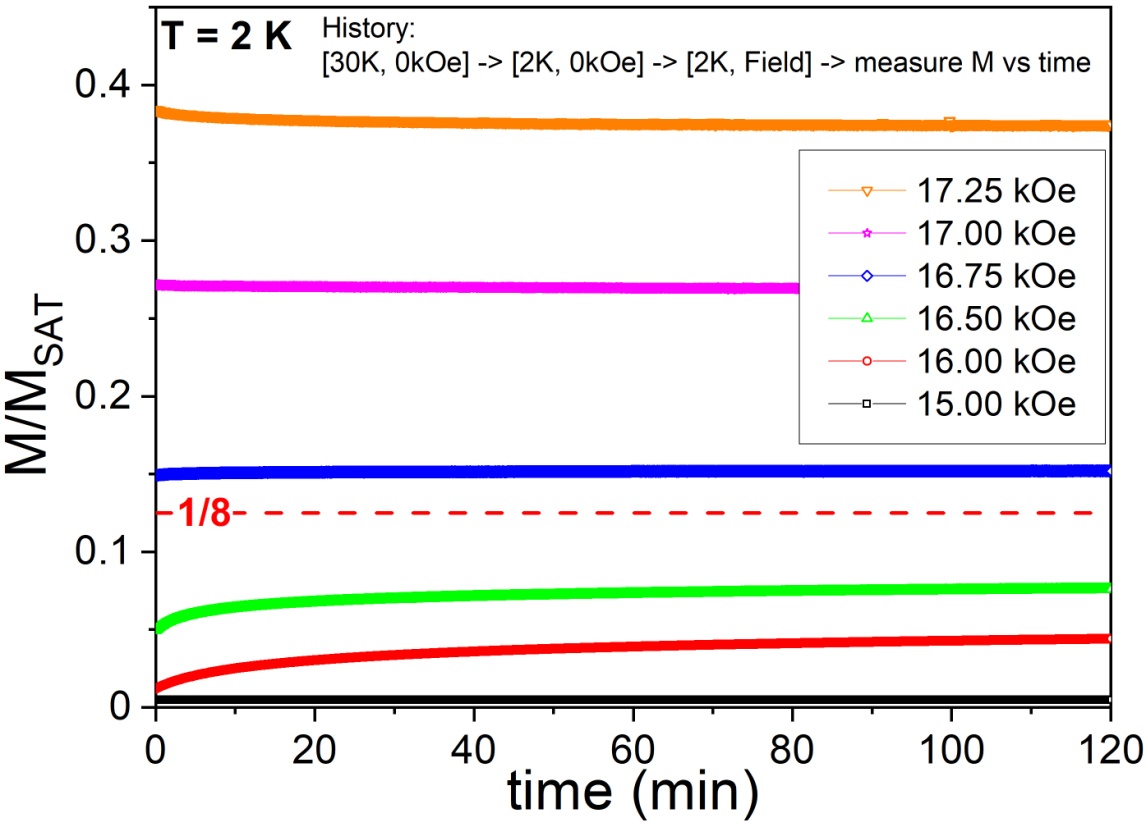


**Fig. S1.** Time dependencies of magnetization after zero-field cooling to 2 K and increasing the field to a value located in the supposed FPP between 15 and 17.25 kOe. The observed dependencies show an initial increase (for *H* < 17 kOe), however, with no clear tendency to reach the *M/M_sat_* = 1/8 fractional plateau.

To see in which *H* - *T* region the new altered AF phase is stabilized, the experimental procedure (shown in Figure 3) was performed at various temperatures between 2 and 5.5 K. For the better visualization of Néel phase alterations at each temperature, the difference between pristine (*M_pristine_*) and “after magnetizing” at 20 kOe (*M_after20kOe_*) magnetization curves is plotted (see Figure S2). One can see that changes of *∆M* appear at 6 kOe for temperatures below ~ 4 K and increase with lowering temperature.


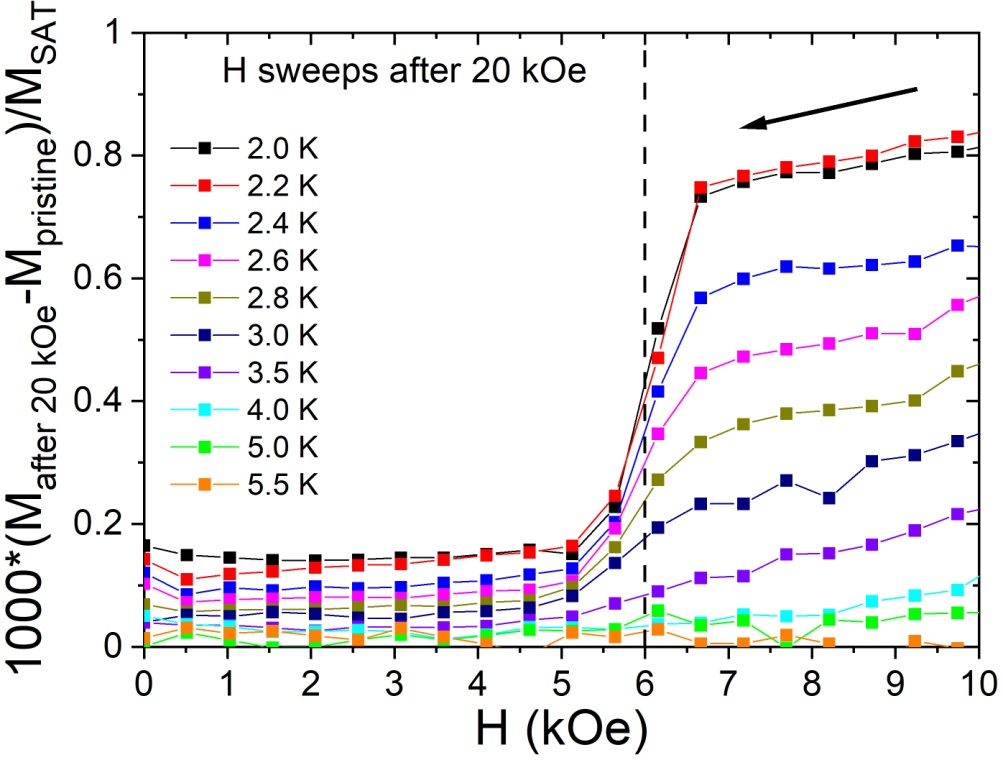


**Fig. S2.** Alteration of the Néel phase magnetization at various temperatures after lowering the field from 20 kOe.

As a reference experiment, a magnetization curve and a corresponding heat capacity dependence were measured by the following way (protocol): (1) - the sample was cooled from 30 K to 2 K in zero field, (2) - then the field *H* was ramped to 50 kOe where *M* = *M_sat_*, (3) - data (either *M* or *C/T*) were collected on ramping *H* down to 0 kOe, (4) - *M* or *C/T* were measured on ramping *H* back up to 50 kOe. The obtained results (see Figure S3) show a qualitatively very good agreement with the work of Trinh et al. [1] which justifies the use of high precision ac-calorimetry.


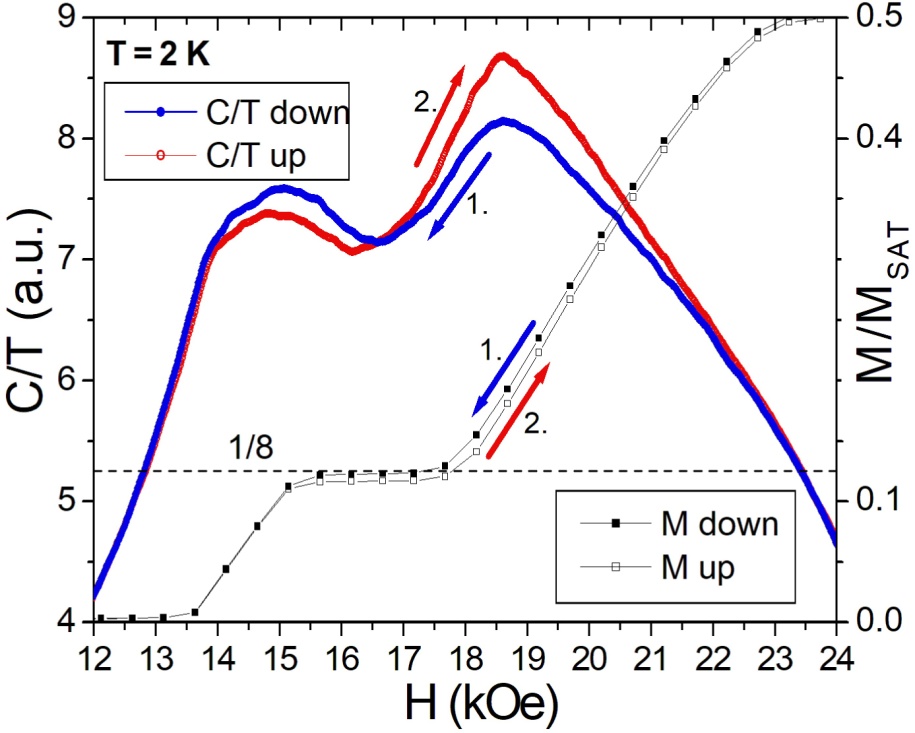


**Fig. S3.** Field dependences of relative heat capacity divided by temperature, *C*/*T* (blue and red line, obtained by ac-calorimetry) and magnetization *M* (black points).

Further it was shown that at states with very low *M*/*M_sat_* values (obtained after annealing), flat plateaus can be observed in field between about 14 and 18 kOe (Figure S4). On the other hand, thermal annealing of the HPP at 25 kOe lead to no changes of magnetization or heat capacity at 2 K, from which also follows that this HP phase is thermodynamically stable and represents a ground state.


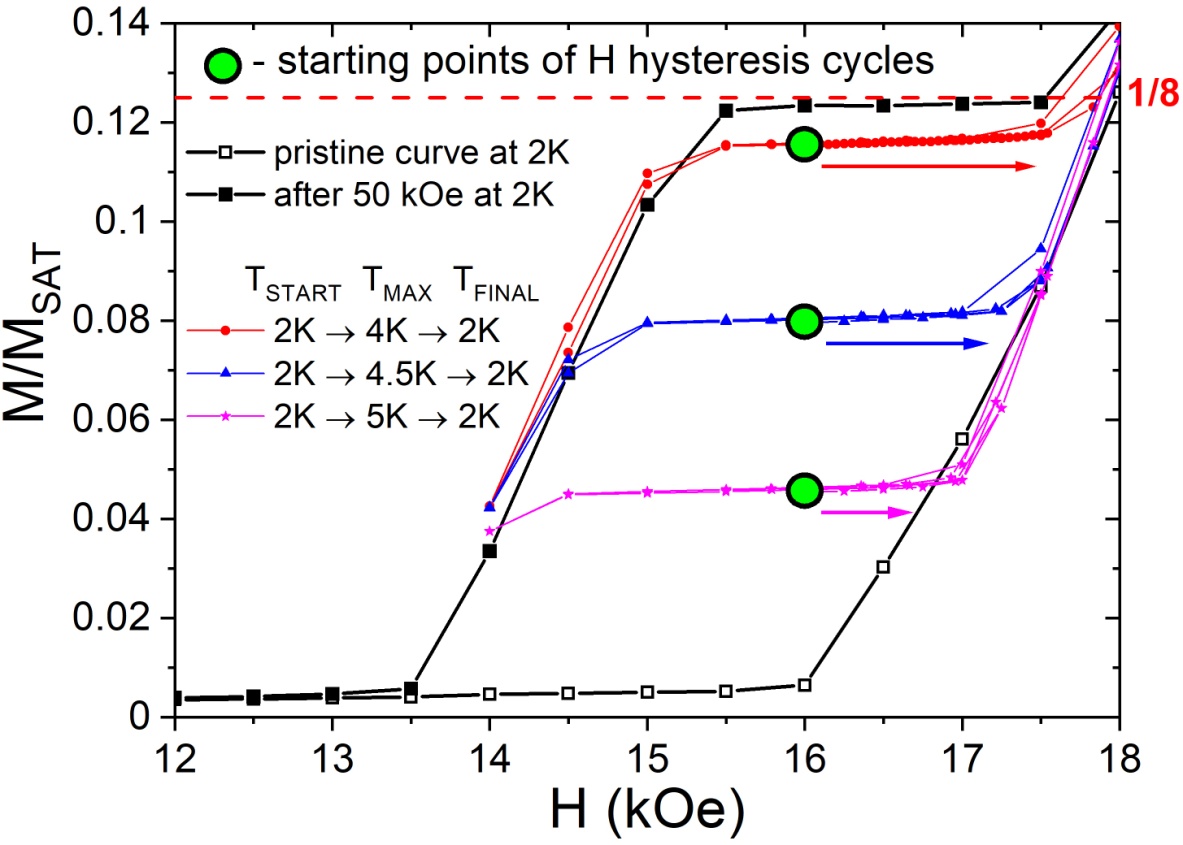


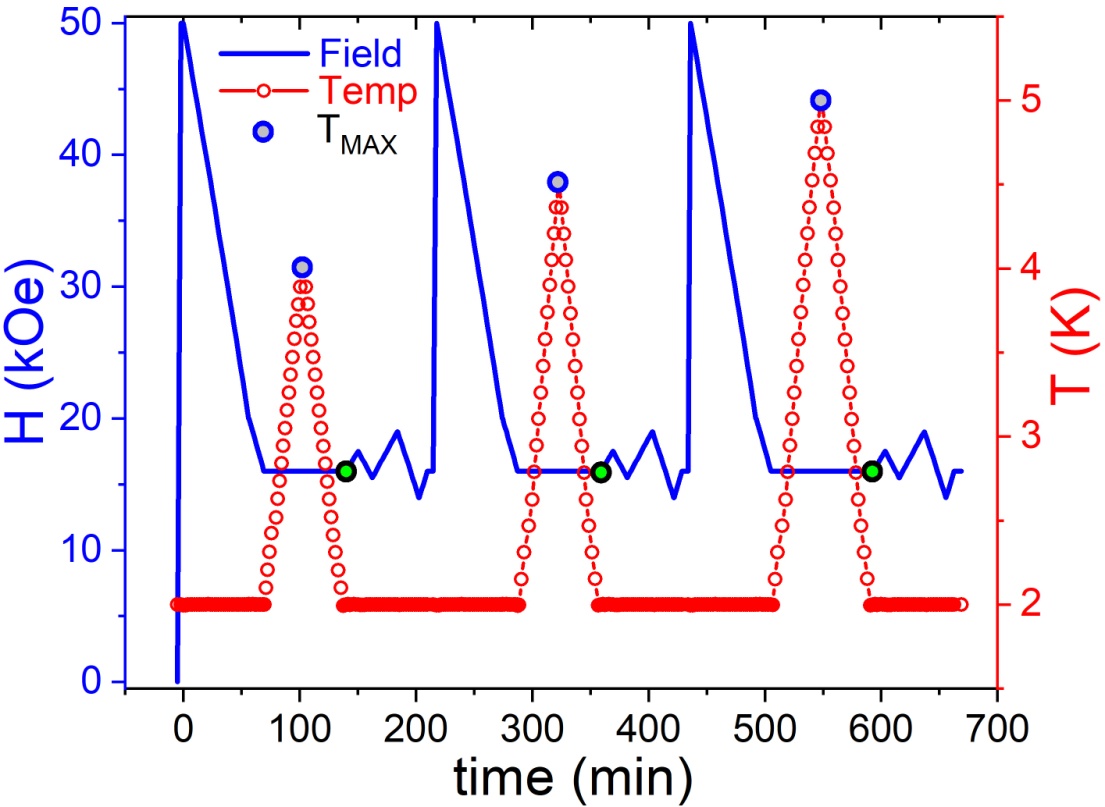


**Fig. S4.** Flat magnetization plateaus at 2 K obtained after annealing to different temperatures *T_max_*. With increase of *T_max_* the value of magnetization plateaus decreases. The detailed field and temperature protocol of performed procedure is shown in the bottom of this figure.

**References:**

1. Trinh, J., Mitra, S., Panagopoulos, C., Kong, T., Canfield, P.C., and Ramirez, A.P. Degeneracy of the 1/8 plateau and antiferromagnetic phases in the Shastry-Sutherland magnet TmB_4_. *Physical Review Letters* **121**, 167203 (2018).
